# Analysis of the goldfish *Carassius auratus *olfactory epithelium transcriptome reveals the presence of numerous non-olfactory GPCR and putative receptors for progestin pheromones

**DOI:** 10.1186/1471-2164-9-429

**Published:** 2008-09-20

**Authors:** Nikolay N Kolmakov, Michael Kube, Richard Reinhardt, Adelino VM Canario

**Affiliations:** 1 Centro de Ciências do Mar, Universidade do Algarve, Campus de Gambelas, 8005-139 Faro, Portugal; 2 MPI Molecular Genetics, Ihnestrasse 63-73, D-14195 Berlin-Dahlem, Germany

## Abstract

**Background:**

The goldfish (*Carassius auratus*) uses steroids and prostaglandins as pheromone cues at different stages of the reproductive cycle to facilitate spawning synchronization. Steroid progestin pheromone binding has been detected in goldfish olfactory membranes but the receptors responsible for this specific binding remain unknown. In order to shed some light on the olfactory epithelium transcriptome and search for possible receptor candidates a large set of EST from this tissue were analysed and compared to and combined with a similar zebrafish (*Danio rerio*) resource.

**Results:**

We generated 4,797 high quality sequences from a normalized cDNA library of the goldfish olfactory epithelium, which were clustered in 3,879 unique sequences, grouped in 668 contigs and 3,211 singletons. BLASTX searches produced 3,243 significant (E-value < e^-10^) hits and Gene Ontology (GO) analysis annotated a further 1,223 of these genes (37.7%). Comparative analysis with zebrafish olfactory epithelium ESTs revealed 1,088 identical unigenes. The transcriptome size of both species was estimated at about 16,400 unigenes, based on the proportion of genes identified involved in Glucose Metabolic Process. Of 124 G-protein coupled receptors identified in the olfactory epithelium of both species, 56 were olfactory receptors. Beta and gamma membrane progestin receptors were also isolated by subcloning of RT-PCR products from both species and an olfactory epithelium specific splice form identified.

**Conclusion:**

The high similarity between the goldfish and zebrafish olfactory systems allowed the creation of a 'cyprinid' olfactory epithelium library estimated to represent circa 70% of the transcriptome. These results are an important resource for the identification of components of signalling pathways involved in olfaction as well as putative targets for pharmacological and histochemical studies. The possible function of the receptors identified in the olfactory system is described. Moreover, the role of olfactory epithelium specific isoforms of classical membrane progestin receptor genes as candidates for preovulatory pheromone sensing is discussed.

## Background

Chemical senses play an important role in aquatic organisms, affecting many aspects of their biology. In teleost fish, social behaviour, reproduction, homing, schooling, search for food and predator avoidance are all regulated by the sense of smell [1-3]. In addition, because of its origin and structure, the olfactory epithelium is the first tissue to be affected by any toxic agent that fish encounter in the natural environment [4,5] and heavy metal accumulation in the fish brain directly affects signal transduction in the central nervous system [6]. However, despite the existence of many physiological and behavioral studies about olfaction in fish, the molecular mechanisms of signal transduction and regulation of olfaction is not fully understood.

It has been proposed that olfactory receptor proteins responsible for odorant recognition belong to the large superfamily of G-protein coupled receptors (GPCR)[7]. Genome-wide analysis of several fish species has provided some data about the number and variability of GPCRs in fish. Indeed, genomic studies of zebrafish (*Danio rerio*), tiger puffer fish (*Takifugu rubripes*) and medaka (*Orizias latipes*) have led to the identification of three main receptor classes possibly involved in olfaction and pheromone communication. These are the principal olfactory receptors (Class A – rhodopsin-like), vomeronasal-type 2 (VT2; Class C – Ca sensing and metabotropic glutamate receptors) as well as a recently described vomeronasal-type 1 homolog class (closely related to Class A) which contains few members [8,9]. The zebrafish genome contains approximately 140 hypothetical Class A receptors, while in puffer fishes there are 44 and approximately 50 Class C receptors [8,9]. Relatively few of the receptors have been isolated as cDNA and some are expressed in tissues other than the olfactory epithelia, raising questions about their exclusive role in olfaction.

Despite the importance of the goldfish (*Carassius auratus*) for behavioural studies and its well characterised pheromonal system [10], only 22 receptors have been cloned so far, 4 from class A and 18 from class C [11-13]. Moreover, for only a single olfactory receptor, OR5.24, has ligand preference been characterised and shown to bind positively charged, amino acids [12]. However, a wide group of chemicals are known to induce a physiological response in the goldfish. Such chemicals include, amino acids and polyamines [14], which are the main signal for recognition of food and danger; bile acids which are involved in social behaviour between conspecifics or related species [15]; steroids, mainly the progestins 17,20β-dihydroxy-4-pregnen-3-one (17,20β-P), 17,20β-P sulphate and androstenedione, which synchronize gonadal maturation among sexes [16,17], and prostaglandin F_2α _(PGF_2α_), which elicits spawning behaviour [18]. It has been shown that steroid- and prostaglandin responses are at least partly mediated by the cyclic AMP signalling pathway [19] and that olfactory epithelium cells have steroid-binding activity [20].

The objective of the present study was to obtain insight into molecular mechanisms of olfactory and in particular pheromone signaling by analysing expressed sequence tags (ESTs) of the goldfish olfactory epithelium. Identification of candidate genes potentially involved in the regulation and structural organization of olfactory signaling pathways in fish should contribute to extend knowledge of receptors and auxiliary proteins underpinning olfaction. To this end an EST collection produced from a normalized cDNA library of the olfactory epithelium from sexually mature goldfish was analysed. Comparison of olfactory epithelium ESTs from goldfish and zebrafish, both cyprinids, allowed an estimation of transcriptome size in the goldfish olfactory epithelium to be made. The presence of splice variants of membrane progestin receptors (PAQR) are reported for the first time in olfactory epithelium together with genes involved in their regulation. A number of new olfactory and other G-protein coupled receptors have been identified as well as a group of Class A subfamily receptors, closely related to *Xenopus *amino acid olfactory receptors.

## Results and Discussion

### Descriptive statistics of Expressed Sequence Tags

A total of 6,144 EST were produced from a normalized goldfish olfactory epithelium cDNA library. After subtraction of poor quality sequences, the average length per EST of the remaining sequences (4,797) was 608 base pairs. The sequences were assembled into 668 contigs and 3,211 singletons (Table 1) [see Additional file 1]. Because further assembling was not possible, the sequences obtained were considered unigenes, each unigene representing the product of a separate gene. Unigenes corresponded to about four-fifths of the total sequences obtained (Table 1). Most of the genes identified were isolated for the first time in this species, highlighting the paucity of gene expression studies in the species. Normalization was highly efficient as demonstrated by a low skewness (4.56) and kurtosis (37.6). Contigs were composed of multiple ESTs with a maximal depth of 11. Only 9 contigs had a depth of more than 5 and the upper 25 percentile contained only 41.4% of all ESTs. By comparison, zebrafish ESTs pooled from two non-normalized olfactory epithelium libraries comprised 6,696 unigenes from 34,699 counts. Pooling of the two zebrafish olfactory epithelium libraries did not significantly change redundancy – skewness of the pooled zebrafish EST libraries was 13.76 versus 13.91 for the average of the two separate libraries, and kurtosis was 233.3 versus 245.4. Maximal contig depth in the pooled libraries was 512 EST counts and the upper 25th percentile contained 80.1% of total EST counts. The percentage of goldfish unigene sequences which had a statistically significant similarity to genes deposited in GenBank was 83.6%, compared to 92.6% for the pooled zebrafish olfactory epithelium unigenes.

**Table 1:** Summary of goldfish EST sequences analysis


Total ESTs sequenced	6144
Sequences passed quality control	4797
Average length per good quality EST (bp)	608
Number of contigs	668
Number of singletons	3211
Total of unigenes	3879
Unigenes with database hits	3243 (83.6%)
Unigenes with a known function	1464 (37.7%)

### Comparison between Goldfish and Zebrafish olfactory epithelium ESTs

**Figure 1:**
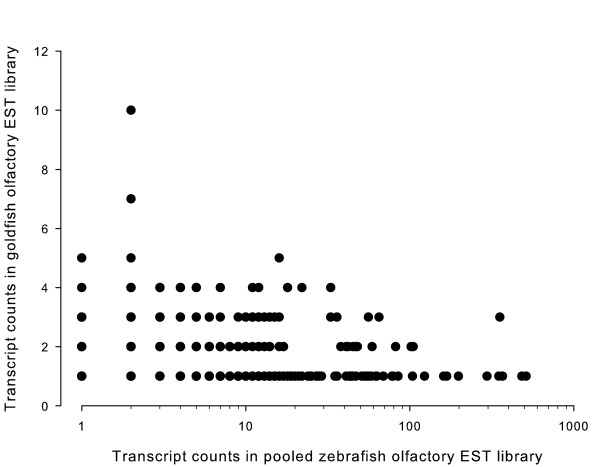
**Frequency of common transcripts between goldfish and zebrafish olfactory EST libraries**. Semi-logarithmic plot of counts of each transcript present in both the goldfish normalized and zebrafish non-normalized EST library. The almost uniform relationship (R = 0.009) is a strong indication of absence of amplification bias during normalization.

The similarity of the goldfish and zebrafish olfactory epithelium EST collections was examined using side-by-side comparison of the total list of identified genes. Unidentified goldfish unigenes were further compared to the pooled zebrafish EST olfactory library using BLASTn with an E-value < e^-40^. From the preceding analysis, a total of 1,088 common unigenes were obtained, of which 44 lacked annotation to known gene products. The goldfish unigenes were evenly represented throughout the frequency distribution range of unigenes in the zebrafish non-normalized library, suggesting representation bias was minimal or absent in the goldfish library (Figure 1). To further substantiate the observed lack of representation bias in the normalized goldfish olfactory epithelium library, one thousand clones were randomly picked and sequenced from a non-normalized, goldfish olfactory epithelium library (not described) and yielded 253 unigenes. Comparative analysis of the 253 unigenes with the goldfish normalized library and zebrafish pooled library revealed 66 and 131 matches, respectively and 104 unigenes which were absent from both libraries. The ratio of goldfish/zebrafish matches in the latter analysis was 50.4%, similar to the results obtained for the normalized goldfish library, further supporting the notion of a lack of amplification bias during the normalization process.

### Transcriptome estimation

In order to obtain an estimate of the goldfish olfactory epithelium transcriptome size, a list of 75 genes associated with Glucose Metabolic Process in animals was extracted from the Gene Ontology (Table 2). Of these genes, 15 (20%) were present in the normalized goldfish olfactory epithelium EST collection and 28 (37.3%) were found in the pooled zebrafish olfactory epithelium library. Extrapolation of the proportion of Glucose Metabolic Process genes in these libraries to the total number of unigenes for each library gave an estimate of the olfactory epithelium transcriptome size of, respectively, 16215 and 16612 unigenes for goldfish and zebrafish. The ratio of Glucose Metabolic Process genes present in the goldfish and zebrafish EST libraries (53.6%) was similar to the ratio of the number of unigenes present in the two libraries (52.3%). This is in agreement with the hypothesis that the transcriptome of this tissue in the two species is of a similar size.

**Table 2:** Unigenes from the Glucose Metabolism category (GO:006006) in goldfish and zebrafish olfactory epithelium collections

*Genes only in goldfish OE transcriptome*	*Genes in OE unigene libraries of both species*	*Genes only in zebrafish OE transcriptome*
cAMP responsive element modulator	Activating transcription factor 4	3-hydroxyisobutyrate dehydrogenase
Hexokinase 1	Aldolase 2, B isoform	6-phosphofructo-2-kinase/fructose-2,6-biphosphatase 3
Nuclear receptor subfamily 3, group C, member 1	Glycerol-3-phosphate dehydrogenase 1 (soluble)	6-phosphogluconolactonase
Phosphofructokinase, platelet	Leptin	Adiponectin, C1Q and collagen domain containing
Ribose 5-phosphate isomerase A (predicted)	Phosphoglucomutase 3	Aldhehyde dehydrogenase family 5, subfamily A1
Solute carrier family 2, (facilitated glucose transporter), member 8	Ribulose-5-phosphate-3-epimerase (predicted)	Calcium channel, voltage-dependent, P/Q type, alpha 1A
Transketolase	Transaldolase 1	cAMP-regulated phosphoprotein 19
	Triosephosphate isomerase 1	Carbonic anhydrase 5a, mitochondrial
		Carnitine palmitoyltransferase 1a, liver
		Enolase 1, alpha
		Fatty acid binding protein 5, epidermal
		Glucose phosphate isomerase 1
		Glucose-6-phosphate dehydrogenase X-linked
		Glyceraldehyde-3-phosphate dehydrogenase, spermatogenic
		Insulin-like growth factor binding protein 1
		Myelocytomatosis oncogene
		Protein kinase, AMP-activated, alpha 1 catalytic subunit
		Pyruvate dehydrogenase (lipoamide) beta
		Pyruvate kinase, muscle
		Tumor necrosis factor

Total number of Glucose Metabolism genes	75	100%
Positive matches to goldfish OE library	15	20%
Positive matches to zebrafish OE library	29	37.3%
Unmatched genes	40	53.3%

Positive matches include counts of genes present in both libraries. For this reason the sum of present and absent genes exceeds the total number of genes used in analysis.

The close phylogenetic relationship between goldfish and zebrafish and the almost identical size of estimated transcriptome of their respective olfactory epithelia led us to combine the ESTs from the two species into a "cyprinid" olfactory epithelium transcriptome. In order to do this identical unigenes were treated as the same, while closely related genes (same gene subfamily, subunits of the same protein complex, etc.) were listed separately. Any information about levels of expression was removed, thus the list contains information only about presence of genes in the olfactory epithelium. As a result, 9,596 unique sequence identifiers were assembled, which comprised 58.5% of the estimated cyprinid olfactory epithelium transcriptome. This percentage is what would be predicted based on the unigenes obtained from the non normalized goldfish olfactory epithelium, in which clones absent from goldfish and zebrafish libraries comprised 41.1% (compared to expected random picking of 41.5%). As a result we estimate that the "cyprinid" olfactory epithelium transcriptome is approximately 16,400 unigenes.

Taken together these data support the use of the "cyprinid" unigene set in further analysis of olfactory epithelium gene expression.

### General description of genes in the transcriptome

Since normalization of the goldfish olfactory epithelium library was highly efficient, it is not possible to use it to determine the level of expression of the most abundant mRNA species using ESTs counts. For this purpose the list of unigenes from the pooled zebrafish library was used. The unigenes with a redundancy level higher than 0.5% are listed in Table 3.

**Table 3:** Genes highly represented in the zebrafish olfactory library


*EST count*		*Gene name*	*Gene description*
		
Percentage	Copies		

1.48	512	*EEF1a*	Elongation factor 1-alpha.*
1.39	482	*ApoE*	Apolipoprotein Eb*
1.38	480	n/a	Unknown protein
1.25	433	*Iclp1*	Invariant chain-like protein 1*
1.20	416	*Actb*	Beta-actin 2
1.14	395	n/a	Zgc:112103
1.06	368	*Slc25a4*	Similar to solute carrier family 25 (mitochondrial carrier; adenine nucleotide translocator), member 4*
1.06	368	*Rplp0*	Ribosomal protein, large, P0*
1.02	354	*Fth1*	Ferritin, heavy polypeptide 1*
1.00	349	*B2m*	Beta-2-microglobulin*
0.98	341	*Tyrp1*	Tyrosinase-related protein 1
0.89	309	*Gnb2l1*	Guanine nucleotide binding protein (G protein), beta polypeptide 2-like 1(RACK1)*
0.85	296	*Bat5*	Similar to HLA-B associated transcript 5, rat orthologue*
0.82	286	n/a	Unknown protein
0.74	257	*Iclp2*	Invariant chain-like protein 2*
0.72	250	*Tpt1*	Similar to translationally-controlled tumor protein
0.67	232	*Rpl4*	Ribosomal protein L4
0.60	208	*Tuba6*	Similar to tubulin alpha 6
0.57	198	*Rpl8*	Ribosomal protein L8*
0.53	185	n/a	Wu:fc48a12*

Twenty most represented ESTs in zebrafish olfactory epithelium. Stars indicate matches to goldfish olfactory epithelium.

The 20 most expressed genes in the olfactory epithelium of adult zebrafish could be divided into four groups: 1) immune response, 2) cell machinery and cytoskeleton, 3) tissue specific function and 4) unidentified. Immune response genes were represented by 4 genes from both MHCI (invariant chain-like protein 1 and 2, HLA-B associated transcript 5) and MHCII (beta-2-microglobulin) [21]. Cell machinery genes included those encoding 3 ribosomal proteins, mitochondrial carrier slc25a4, beta-actin, tubulin, tubulin-related tctp (*tpt1*) and iron-sequestering ferritin [22-24]. Also in this group elongation factor 1-alpha, related to protein synthesis in general, was most abundant. Among genes generally highly expressed in epithelial or neuronal tissue and expected to have a high level of expression in olfactory epithelium, were apolipoprotein E, tyrosinase-related protein 1 and G-protein beta subunit homologue RACK1. Apolipoprotein E plays an important role in lipid transport and lipoprotein metabolism, both processes involved in the formation of the olfactory epithelium mucosa [25]. The gene for tyrosinase-related protein 1 is involved in pigmentation and melanin synthesis [26]. GNB2L1(RACK1) is a key element of cell migration and adhesion through integrins and IGF and is highly expressed in epithelial cells and fibroblasts [27]. Twelve of the most abundant zebrafish transcripts were also identified in goldfish olfactory epithelium, including the four of immune response genes, apolipoprotein E and G-protein beta subunit homologue. A recent analysis of a small set of channel catfish (*Ictalurus puntactus*) olfactory epithelium ESTs reported by Li et al. [28] also described the presence of immune system genes, including invariant chain-like proteins, cytokines and lymphocyte markers, and some other non-neuronal elements also identified in goldfish and zebrafish by our analysis. The expression of these genes in independently produced cDNA libraries of the olfactory system of the three species suggests it is not due to possible contamination with other tissues and corresponds to the expectation of a well developed specific immune barrier, necessary to protect the unique neuronal tract, which directly links the brain with environment.

**Figure 2:**
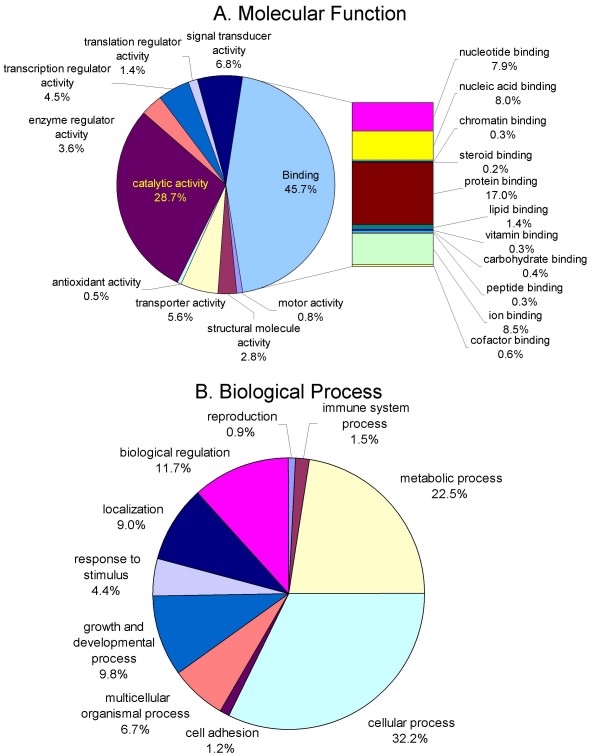
**Classification of EST according to Gene Ontology**. Distribution of goldfish olfactory epithelium unigenes classified using GeneOntology categories of (A) Molecular Function and (B) Biological Process.

The unigenes of the goldfish olfactory epithelium library with identity to known gene products were assigned to Gene Ontology (GO) categories using data from Gene Ontology and GeneCards databases. A total of 1,464 unigenes was successfully assigned to 10,335 GO categories. Within the Molecular Function category, most of the identified proteins were involved in binding (45.7%), catalytic activity (28.7%), transporter (5.6%), and signal transducer activity (6.9%). In terms of physiological processes, the most represented unigenes correspond to cellular process (29.8%), metabolism (20.9%), and biological regulation (11.7%) (Figure 2). When comparing with gene ontology categories of channel catfish gill epithelium [28] some specificities become evident. For example, the representation of both transcription regulatory mechanisms and DNA binding proteins in gill is proportionally twice that of the olfactory epithelium possibly because of a higher diversity of cell types (e.g., cartilage, bone, different types of epithelial cells) in gills. In contrast, at the translational level the olfactory epithelium has a higher proportion of regulatory elements and protein binding representatives. Also, within the binding category in olfactory epithelium there were some elements, absent from gill, possibly related to the tissues sensory function, such as over-represented genes involved in nucleotide (mainly cAMP) and ion (Ca^2+^, Na^+ ^and Cl^-^) binding. Furthermore, comparison of Biological Processes revealed a higher proportion of genes involved in growth, development and apoptotic mechanisms in the olfactory epithelium possibly reflecting intensive recirculation of the neurons.

### GPCR receptor repertoire in the olfactory epithelium

#### Class I GPCRs – Rhodopsin family

Several dozen G-protein coupled receptors were identified in the fish olfactory epithelium tissue as one or more EST copies, the majority belonged to Class I or the Rhodopsin family. This class could be further subdivided into three major subgroups: 1) immune system related receptors, 2) receptors to purines, pirimidines, and hormones, as well as neuromediator-related receptors and 3) olfactory receptors and orphan GPCRs.

##### GPCRs related to the immune system

This subgroup (Table 4) includes GPCRs containing the C-C motif chemokine receptors 5 and 6 (ccr*), C-X-C motif receptors 3.2, 4, 5 and 7 (cxcr*), leucotiene B4 receptor 2 (*ltb4r2*), GPRs 34b, 63 and 132. The Epstein-Barr-virus-induced G-protein coupled receptor 2 (*ebi2*) is also part of this subgroup. *CCR5 *is expressed in lymphoid organs such as thymus and spleen, as well as in peripheral blood leukocytes, including macrophages and T cells, and stimulates leukocyte chemotactic activity through generation of inositol phosphates in the presence of macrophage inflammatory protein 1 [29]. *CCR6 *expression is restricted to functionally mature memory B cells, capable of responding to antigen challenge [30]. Splice variant 2 of *CXCR3 *has been shown to play a key role in transduction of the angiostatic effect of cytokines in microvascular endothelial cells [31]. Chemokine receptor 4 (*cxcr4*) affects cell-cycle proteins in hippocampal or cerebellar granule neurons and through cytokine binding represses the activity of E2F-dependent apoptotic genes to maintain neurons in a highly differentiated and quiescent state [32]. The ligand CXCL12 is a main axon guidance agent in the zebrafish olfactory system [33]. CXCR7 is a co-receptor for CXCR4 in B-, T-cells and monocytes [34]. CXCR5 is a cytokine receptor involved in B-cell activation and chemoattraction [35]. *GPR132 *is predominantly expressed in lymphocytes and macrophages where it serves as a mitosis delay mechanism under control of lysophosphatidylcholine and endogenous lysophosphatidic acid DNA damage signals [36]. GPR34 is the receptor for lysophosphatidylserine in mast cells. It is reported to be expressed in brain and adipose tissue and is involved in wound healing [37]. Endothelial differentiation G-protein coupled receptor 5 (*Edg5*) is a receptor for sphingosine 1-phosphate, which is responsible for neurite retraction and also keratinocyte growth arrest and differentiation during wound healing [38]. G protein-coupled receptor 63 (*GPR63*) is homologous to *Xenopus *PSP24β [39] which is expressed mainly in neuronal cells, such as olfactory mitral cells, cortical neurons, hippocampus pyramidal cells, and Purkinje cells in the cerebellum. Preliminary studies proposed sphingosine-1-phosphate as the ligand and G-protein αq (Gαq) as an effector for this receptor [40]. Leukotriene B(4) receptor 2 is expressed in mononuclear lymphocytes and provokes chemotaxis in a pertussis toxin-sensitive manner, relating its action to activation of the Gαi pathway [41]. GPR81 is an orphan receptor, similar to neurotensin receptor, which is exclusively expressed in T-cells [42]. EBV-induced G-protein coupled receptor 2(*ebi2*) is most closely related to the thrombin receptor. It is expressed in B-lymphocyte cell lines, lymphoid tissues and is also expressed at lower levels in a promyelocytic (glial precursor) cell line [43].

**Table 4:** Known function of immune system GPCRs


*Cytokine receptors*	
Chemokine (C-C motif) receptor 5	Stimulates leukocyte chemotactic activity
Chemokine (C-C motif) receptor 6	Associated with mature memory B cells
Chemokine (C-X-C motif) receptor 3 splice 2	Transduction of angiostatic effect of cytokines in microvascular endothelial cells
Chemokine (C-X-C motif) receptor 4	Neuronal survival; maintaining of highly differentiated neurons; axon guidance
Chemokine (C-X-C motif) receptor 7	Co-receptor for CXCR4 in B-, T-cells and monocytes
Chemokine (C-X-C motif) receptor 5	B-cell activation and chemoattraction
*Non-cytokine receptors*	
EBV-induced G-protein coupled receptor 2	B-lymphocyte and promyelocytic (glial precursor) cell line regulation
GPR34	Lysophosphatidylserine receptor in mast cells; wound healing
GPR81	Exclusively expressed in T-cells; neurotensin-related
GPR132	Mitosis delay under control of lysophosphatidylcholine in lymphocytes and macrophages
Leukotriene B(4) receptor 2	Chemotaxis of mononuclear lymphocytes
Prostaglandin E2 receptor EP4 (PTGER4)	Stimulates immune response in B-cells and monocytes
P2Y10 (putative nucleotide receptor)	Monocyte development

GPCRs related to immune system expressed in fish olfactory epithelium.

Expression of such a comprehensive immune repertoire is puzzling as immune cells are generally of low abundance in the olfactory epithelium and have been reported in the basal layer of epithelium, as shown in *Labeo rohita *[44]. One possible explanation could be that cytokines, in addition to their role as mediators of the immune response are also involved in nervous system development and neuronal differentiation [45]. Moreover, as significant proliferation of neurons takes place in the olfactory system elements of the immune system may be part of a selective internal mechanisms of sensory cell population regulation via apoptotic mechanisms [46]. An insight into the possible role for different types of immune cells, as well as astroglia, in this process may be derived from multiple sclerosis models, where de-myelination and neuronal degradation mechanisms have been shown [47].

##### GPCRs for purines, pirimidines, hormones and neuromediators

This subgroup includes P2Y5, 6, 7, 10; 5-HT (serotonin) receptor F1, beta-3b-adrenergic receptor, prostaglandin E2 receptor EP4 (*ptger4*), gastrin-releasing peptide (bombesin) receptor and histamine receptor H2.

A ligand for purinergic receptor 5 (*p2ry5*) still has to be identified [48]. Pyrimidinergic receptor P2Y6 (*p2ry6*) is expressed in human nasal epithelial cells and activates the IP3 signaling pathway in the presence of UDP, and regulates mucin and fluid secretion [49]. P2RY7 is a leukotriene B(4) receptor, regulated by GRK6 and is responsible for extracellular ATP-dependent regulation of L-type Ca^2+ ^channels. P2Y10 has so far not been identified as a nucleotide activated receptor, but has been shown to be involved in monocyte development [48]. While the exact cell type, expressing nucleotide receptors in fish is still unknown, there is the possibility that they can be involved in olfactory response modulation in response to noxious odorants, as shown in mice [50]. They could also be immediate olfactory receptors, since the fish olfactory epithelium is able to sense several nucleotides [51].

5-HT1F is a sumatriptan-sensitive serotonin receptor shown to be preferentially expressed in trigeminal ganglia and brain blood vessels where it plays role in regulation of migraine development [52]. It is also a potent inhibitor of olfactory responses in goldfish (Kolmakov et al, personal observation). The recently discovered trace amine-associated receptors (TAAR) [53] are also expressed in the cyprinid olfactory epithelium, with only one homologue each of *TAAR9 *and *TAAR3 *identified in goldfish. At least 24 TAAR ESTs have been identified in zebrafish olfactory epithelium [54], although 57 intact genes have been reported to be present in the genome [55]. Based on the 17 TAAR transcripts present in our "cyprinid" olfactory library we suggest approximately 40–45 TAAR genes are expressed in the olfactory epithelium of cyprinids. Although a role for TAAR receptors in mammalian olfaction of pheromonal volatile amines has been reported, there is still no direct evidence for a role in fish olfaction [54]. However, the expression of TAAR homologues in sea lamprey (*Petromyzon marinus*) olfactory epithelium is supportive of the notion that TAARs are an ancient group of olfactory receptors [56]. Perhaps their close phylogenetic relationship to biogenic amine receptors may indicate that in fish they serve, together with 5-HT1F, as receptors for catecholamines, potent odorants in goldfish [57]. Another possibility could be a role as part of a regulatory signal transduction mechanism with ligands like lysophosphatidic acid and sphingosine, similar to a group of the phylogenetically closely related endothelial differentiation G-protein coupled receptors (EDGs), also expressed in the olfactory epithelium.

The prostaglandin E_2 _receptor EP4 (*ptger4*) is an effector of PGE_2 _inhibition of bone-resorbing activity in functionally mature osteoclasts [58]. It also stimulates the immune response in B-cells and monocytes [59]. An EST was isolated sharing similarity only to the second translated exon of *ptger4*, while the N-terminus did not give any match to the zebrafish genome or any other database. Taking into account that in most prostaglandin receptors the first translated exon encodes 6 of the 7 transmembrane helices, including the putative ligand-binding pocket [60], and that they share a high level of sequence similarity in the second translated exon, this new cDNA could be a new receptor, probably still from the tromboxane/prostaglandin family. It will be of interest in the future to determine if it is a candidate receptor for PGF_2α _the postovulatory pheromone in goldfish.

The beta-3b-adrenergic receptor, together with the beta-2-adrenoreceptor are able to form heterodimers with other GPCRs of class A, altering their ligand specificity, signaling pathway and internalization [61,62]. In mice, the beta-2-adrenoreceptor co-expresses with olfactory receptors and is necessary for their successful translocation to the cell surface [63]. Finally, the bombesin receptor is involved in synaptic plasticity regulation in brain, as well as regulation of a broad spectra of behaviors including feeding and social interaction [64] while the histamine receptor is shown to be important for activation of astrocytes in neonatal rat brain [65].

##### Olfactory receptors and orphan GPCRs

This subgroup includes GPR108 and its homologue TMEM87B both members of the LUSTR family of proteins containing a carboxy-terminal seven transmembrane domain. They are expressed in fungiform papillae in humans and are suggested to be taste receptors [66]. Orphan GPR137 shares identity with a prostate-specific odorant-like GPCR-encoding gene (*PSGR*) and was detected mainly in hippocampus [67]. Orphan *GPR161 *is expressed in rat brain and olfactory epithelium and at a lower level in liver [68]. *GPR173 *is member 3 of the so called "Super Conserved Receptor Expressed in Brain" SREB family, which is highly conserved amongst vertebrates. *SREB3 *member is predominantly expressed in the brain, mainly cerebellum, and ovaries, but not in testis, and remains an orphan receptor [69].

Twelve putative ORs were identified in the goldfish olfactory epithelium EST collection. Based upon the classification of Alioto and Ngai [8], the receptors were assigned to several families: family A members 112-1 and 113-1; family C member 102-1; family D members 107-1, 110-1, 111-10; family E members 125-1, 127-1, 128-5 and three of family F member 115-1 homologues. The average similarity to zebrafish OR was higher than 80% for all the identified goldfish cDNA. Based on the predicted transcriptome size, it seems probable that 45–50 rhodopsin-like olfactory receptors are expressed in the goldfish olfactory epithelium. *OR107-1 *is a zebrafish homologue of the previously cloned goldfish receptor *GFA2 *[11]. Other goldfish olfactory receptors also present in the EST have previously been isolated in families C (*GFA12*, *GFA25*, *CaOR45*) and E (*GFA28*) [11]. Family C is closely related to long-chain non polar aminoacid olfactory receptors of *Xenopus tropicalis*, XB107, 238, 239 and 242 [70]. Two of the members of this family share the same correlated mutational analysis patterns, a strong reason to suspect similarity of ligands [71] making family members good candidate olfactory receptors for methionine, valine, leucine and cysteine.

Additional olfactory receptors were identified by RT-PCR, including receptors from families C, D, E and F and a homologue of the single member of family B (101-1). The total number of confirmed OR genes expressed in the goldfish olfactory epithelium when the results of RT-PCR are taken together with the EST analysis is 41, which closely matches the predicted number of ORs. Surprisingly, neither the goldfish EST annotation, nor RT-PCR were able to identify members of the proposed families G and H, while in the zebrafish olfactory epithelium only a single receptor from family G and 6 receptors from family H have been identified. Also and searches through pufferfish, stickleback and medaka genomes failed to find any family G member which until appears to be restricted only to the zebrafish genome [8]. This may reflect selective adaptation of the zebrafish olfactory system to environmental conditions related to a species specific duplication even and is supported by the fact that the largest cluster of TAAR genes [72] is located less than 2 megabases away on the same chromosome and those genes are also specific for zebrafish.

#### Class II GPCRs – Secretin family

A number of transcripts belonging to the second class of GPCRs (Secretin/VIP family) were identified and include, calcitonin receptor-like receptor (*calcrl*), cadherin EGF LAG receptor 1b (*flamingo*), *GPR64 *and pituitary adenylate cyclase activating peptide (PACAP) type I receptor (*adcyap1r1*). Calcitonin receptor-like receptor has been shown to be co-expressed with RAMP1 in cortex, caudate putamen and olfactory tubercles, providing selective specificity to calcitonin gene-related peptide (*CGRP*) and regulation of endothelial cell proliferation [73]. *Flamingo *is a key receptor regulating dendritic net development in the retina and is also involved in axon guidance [74]. *GPR64 *is an orphan receptor, extensively expressed in epididymis, where it regulates fluid resorption from the ducts and when knocked-out in mice causes male infertility [75]. PACAP type 1 receptor is mainly expressed in the hypothalamus, where it regulates the release of a number of different hormones, including luteinizing hormone, growth hormone and prolactin, and stimulates proliferation of neuronal stem cells upon PACAP activation in mouse [76].

#### Class III GPCRs – γ-Aminobutyric acid (GABA) B/metabotropic glutamate (mGluR) family

The third class of GPCRs identified in olfactory epithelium, GABA (B) and mGluR receptor family includes Ca-sensing receptor, taste receptors of the T1R family and putative pheromone V2R family [9]. The goldfish olfactory library contained ESTs encoding GABA(B) receptor 1 and putative pheromone receptors Cppr10, 11 and GFB 9 and 14. The zebrafish collection contained additionally two homologues of *cppr10*, *cppr1*, *V2R2 *(*GFB8*), two homologues of *GFB7*, *OR5.24 *(*gprc6a*) and orphan *gprc5c*. In the rat olfactory bulb GABA-B receptors couple to Gαi/o and stimulate an increase of cAMP via βγ-mediated activation of adenylate cyclase 2, while simultaneously inhibiting Gαs-mediated activation of other adenylate cyclases [77].

The function of the two metabotropic glutamate receptor ESTs (*mGlur1 *and *mGlur3*) isolated is unclear but electrophysiological studies in the channel catfish indicated that they are directly involved in olfaction of glutamate [78]. Cpprs are putative pheromone receptors of unrevealed cyprinid species from the NCBI database. GFBs were isolated from goldfish, with numbers 9 and 14 represented by full-length cDNA sequences and the rest by partial protein fragments [11]. Odorant receptor 5.24 was first found in goldfish and is the only deorfanized receptor of this family with broad selectivity for positively charged amino acids. Mutagenesis studies of *OR5.24 *revealed inversion from positive to negative charge selectivity by some point mutations [79], the resulting modified sequence is present in related receptors suggesting they are candidate ORs for charged amino acid olfaction. GPRC5C is an orfan receptor from the mGluR family, which has been shown to be expressed under all-trans retinoic acid induction in peripheral tissues, although its function remains to be established [80].

### Progestin receptors in the olfactory epithelium

As previously stated, goldfish and other cyprinids are able to smell progestin steroids, namely17,20β-P and its sulphate and glucuronide conjugates which act as pre-ovulatory pheromones [81]. Physiological cross-adaptation studies of the olfactory epithelium suggest that there are at least two distinctive receptors, one shared by 17,20β-P and its glucuronide and the other for the sulphate [81]. Specific binding for 17,20β-P has been found in olfactory epithelium membrane preparations using radio-receptor assays. [20]. In addition, pharmacological studies suggest that progestin signal transduction in the olfactory system acts *via *activation of the G_αi _(pertussis toxin sensitive) proteins and consequent reduction of cAMP levels [19]. Overall, this physiological evidence is consistent with progestin pheromone receptors belonging to the GPCR family.

Possible candidates for progestin pheromone receptors are among the group of membrane progestin receptors (mPRs), which were first isolated from spotted seatrout (*Cynoscion nebulosus*) oocyte membranes [82] and, more recently, in other fish species, such as the zebrafish (*Danio rerio*), catfish (*Ictalurus punctatus*), medaka (*Oryzias latipes*) and goldfish (*Carassius auratus*) [83,84]. Homologous genes are also expressed in higher vertebrates, from frogs (*Xenopus tropicalis*) to rodents and humans [85]. These genes were identified as members of the progestin and AdipoQ receptor family (PAQR), an ancient group of GPCRs which has evolved from Eubacteria independent of other seven transmembrane domain receptors [86]. Three members of the PAQR family can bind progestins; *PAQR7 *(mPRα), *PAQR8 *(mPRβ) and *PAQR5 *(mPRγ) [85]. As a result of fish specific duplication events, paralogous pairs of all three receptors have arisen. While, both mPRα and β are products from intronless genes, mPRγ is the product of a gene containing eight exons. Furthermore, each gene has distinctive tissue distribution patterns. While mPRα and mPRβ are mainly gonad and brain specific, mPRγ is expressed at higher levels in excretory tissues such as the intestine, colon, kidney and gills [83]. Functionally, they also play distinct roles. For example, mPRβ in ovaries is involved in vitellogenesis but not in final oocyte maturation, as its mRNA expression is unaffected by hormonal stimulation and sharply decreases at late vitellogenesis [87]. In the nervous system, mPRβ expression is related to its role in mediating the stimulatory effects of progesterone in myelin sheath formation and initiation of neuro-protective gene expression [88,89]. In cell lines, mPRβ has been shown to activate the MAP kinase signaling pathway and thereby to regulate gene expression but it has no apparent effect on G_αi_-mediated pathways [84].

The main functions of mPRα in fish are related to reproduction; more specifically, to final oocyte or spermatozoa maturation. In goldfish and seatrout, its expression reaches a peak just prior to final maturation of the oocytes. This peak is necessary for maturation competence under gonadotrophin treatment mediated by the related progestins 17,20β-P and 17,20β,21-trihydroxy-4-pregnen-3-one, respectively [90]. The latter progestin also acts *via *mPRα to stimulate sperm motility in the Atlantic croaker *Micropogonias undulatus *[91]. In the zebrafish, activation of mPRα inhibits adenylate cyclase activity, in a pertussis toxin sensitive manner, implying involvement of G_αi_. The functions of mPRγ are still unclear, but it has been shown to bind progesterone and its hydroxylated derivatives in humans [85].

Genebank contains a number of cDNA for the mPR family and four gene products have been isolated from goldfish ovaries: mPRα (accession number AB122087.1), mPRβ (AB284131.1), mPRγ-2 (AB284133.1) and mPRγ-1 (AB284132.1). Zebrafish mPRα (AY149121.1) and mPRβ (AY149120.1) were reported by Zhu et al. [85] while two orthologues of mPRγ-1, PAQR5a (BC045864.1) and PAQR5b (BC078202.1), are part of the National Institutes of Health Mammalian Gene Collection [92]. mPRγ-2 is a fish orthologue of mammalian PAQR6 and zebrafish 'LOC570587' (NW_001877690.1) was predicted from the genomic sequence.

In the present study several mPR receptors were identified in goldfish and zebrafish olfactory epithelia. In the zebrafish, a single EST for mPRβ and 11 ESTs encoding PAQR5b were identified. In contrast, the goldfish EST library did not contain any of these genes. However, other PAQR family members were identified; adiponectin receptors 1b (*adipor1*) and 2 (*adipor2*) which are involved in lipid metabolism and PAQR3 (Raf kinase inhibitor) which prevents tumorogenesis [93]. RT-PCR studies with zebrafish olfactory epithelium and mPR specific primers revealed two splice variants of PAQR5b. In goldfish olfactory epithelium three different amplicons were obtained, a mPRγ-1 and two splice variants of mPRγ-2. The presence of mPRβ was also confirmed by RT-PCR in the olfactory epithelium of both species, but it was not possible to amplify mPRα.

**Figure 3:**
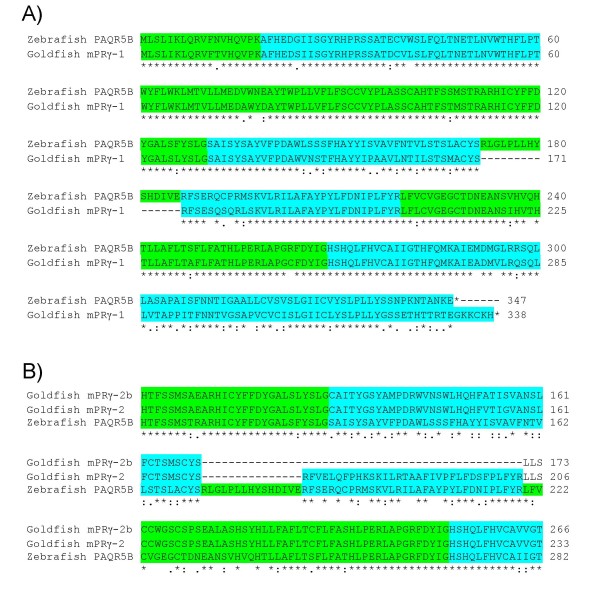
**Alignment of membrane progestin receptor splice variants from the fish olfactory epithelium**. Exon composition of PAQR5b and mPRγ-1 (A, alternating colours) and alignment of two mPRγ-2 splice variants from goldfish olfactory epithelium (B, colours represent corresponding exons 5 and 6 in PAQR5b).

Further analysis of these mPRγ transcripts revealed some interesting properties of one of the splice forms. Between the two paralogous genes, PAQR5a and PAQR5b, the latter is the closest orthologue to the goldfish mPRγ-1, with sequence similarity at both DNA and amino acid sequence levels of about 84% (Figure 3A). The PAQR5b gene product, isolated from the ovaries, is a transcript of the complete set of eight exons. One of the PCR products from the zebrafish olfactory epithelium represented exactly the same splice variant. Another PAQR5b PCR product and all 11 EST sequences from the zebrafish cDNA library are the result of alternative splicing, where exon number 5, a 15 amino acid fragment, is absent. The mPRγ-1, reported earlier in ovaries and isolated in the current study from goldfish olfactory epithelium, lacks the same fragment (Figure 3B). ESTs of mPRγ-1 paralogue, containing exon 5, from other fish species are also available in GenBank: the phylogenetically distant stickleback (*Gasterosteus aculeatus*; e.g. DN666595), as well as other cyprinids such as the roach (*Rutilus rutilus*; EG532701) and fathead minnow (*Pimephales promelas*; DT191559). However, it was not possible to establish if the lack of exon 5 is a splice variant in goldfish or if this exon has been lost during evolution in this species.

**Figure 4:**
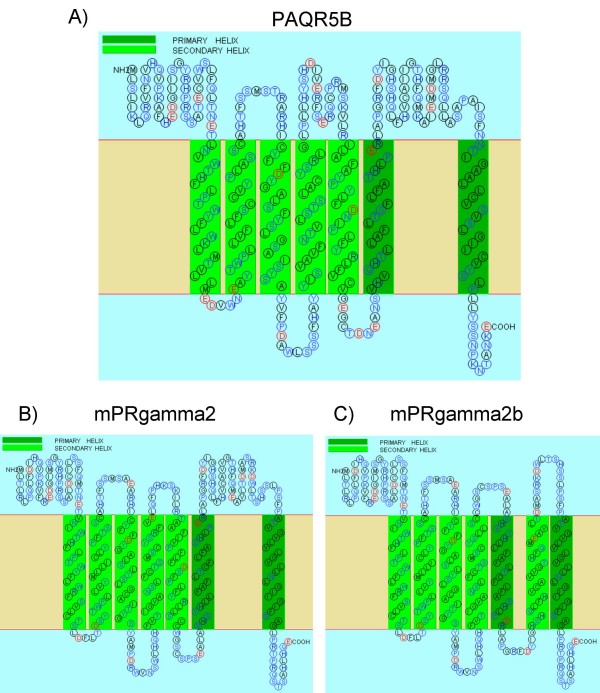
**Predicted secondary structure of membrane progestin receptors from the fish olfactory epithelium**. Snake-plot of complete PAQR5b and two mPRγ-2 splice variants from fish olfactory epithelia built using SOSUI prediction. Note that TM architecture remains despite significant rearrangement of transmembrane helices in case of mPRγ2b.

Two other PCR products from goldfish were splice variants of mPRγ-2. Whilst overall homology between protein sequence of mPRγ-2 and PAQR5b was only 55%, the amino acids on the borders of exons are well conserved and the gene organization of PAQR6 and PAQR5 is identical. This allowed alignment of PCR products against PAQR5b to establish exon composition and distinguish between splice variants and identify missing exons (Figure 3B). One of the PCR products amplified was identical to previously described mPRγ-2 from ovaries. This transcript resembles mPRγ-1 in that it also lacks exon 5. A novel splice variant was identified which lacks the relatively short exon 5 and the longer (33 amino acids) exon 6 (Figure 3B). Using a combination of SOSUI [94] and TMpred [95], the putative trans-membrane domain combinations for PAQR5b, mPRγ-2 (lacking exon 5) and mPRγ-2b (lacking exons 5 and 6) (Figure 4) was predicted. This analysis revealed that exon 5 encodes part of the second extra-cellular loop and its loss does not radically change the configuration of the trans-membrane domains. However, in the splice variant missing both exon 5 and 6, complete re-arrangement of trans-membrane domains 5 and 6, as well as the third extra-cellular loop may occur (Figure 4C). We predict that the initial domain number 6 becomes inverted taking the place of the absent domain number 5 and that part of the long third extra-cellular loop, containing a low-score alpha helix, becomes the new domain 6, preserving the stable seven trans-membrane architecture. Nevertheless, it is likely that such a drastic change in extra-cellular and trans-membrane architecture changes the ligand-binding properties and, possibly, the transduction pathway.

Screening of the NCBI EST database for the identified splice variants revealed a complete form of PAQR5b is expressed in the intestine, trunk kidney, colon, gills and gonads of fish. Single EST copies for the mPRγ-1-like splice variant were present in head kidney, skin and fins but the majority were located in zebrafish olfactory epithelium. mPRγ-2 was found only at low levels in the brain of zebrafish and the fathead minnow. However, in an ancient vertebrate, the sea lamprey (*Petromyzon marinus*), a single EST (EE741072.1) was identified for this receptor in the olfactory epithelium. In mammals, this receptor is expressed only in brain areas which have connections to the accessory olfactory bulb, suggesting a role in pheromonal signal processing. No sequence which lacked both exons 5 and 6 was identified in any sequence database and none of these genes were found in the mammalian olfactory system or in frog olfactory bulb.

Taken together, these results demonstrate the presence of several membrane-associated G-protein coupled receptors specific for progestin pheromones in the olfactory epithelium of goldfish and zebrafish. According to physiological and biochemical studies, GPCR pheromone receptors function *via *G_αi _[20]. As mPRα was not identified in either species and activation of mPRβ does not decrease cAMP, mPRγ appears to be a good candidate for progestin-related signaling in the olfactory system. Several splice forms of the receptor were isolated in both species, one of which appears be unique to the goldfish olfactory epithelium, while another was over-represented in zebrafish olfactory epithelium compared to other tissues. The absence of mPRγ in the tetrapod olfactory system, its presence in early vertebrates and its affinity for hydroxyl-containing progesterone derivatives (but not estrogens, androgens or glucocorticoids) suggests a specific role for these gene products in detecting pheromonal progestins in some fish. In conclusion, we propose PAQR5 and PAQR6 (mPRγ-1 and mPRγ-2 in goldfish) as progestin pheromone receptors in cyprinids. However, further studies are necessary to confirm or discard this hypothesis.

## Conclusion

Through comparisons and combination of the goldfish olfactory epithelium ESTs produced in this study with those available for zebrafish it was possible to estimate that the olfactory epithelium transcriptome contain around 16,400 unigenes and to identify a number of nervous tissue markers and several elements of signalling pathways involved in olfaction. For example, 124 GPCRs were identified and included receptors for cytokines, neurotransmitters, growth and development factors, hormones, steroids and olfactory cues.

The presence of a large number of immune system genes may reflect the epithelial character of the tissue and also the necessity for tight control on intensive processes such as, regeneration of the neuron population which involves cytokines and immune cells. Hormonal receptors also help us to understand regulatory mechanisms involved in the functioning and precise adjustment of the olfactory system. Growth factor receptors provide an important tool for developing selective markers for the different cell types composing this complex tissue and to study processes involved in differentiation of sensory neurons. The TAAR receptors found in the goldfish olfactory library were not as diverse as expected from genomic prediction and expression studies in zebrafish, raising the possibility of species-specific adaptations in the olfactory system.

One third of the GPCR repertoire was classical ORs and indicates that fish probably recognize a wide variety of potential odorants. While most ORs remain orphans, two subfamilies were linked to specific amino acid subclasses, long-chain non polar and charged. Receptors known to bind nucleotides were also identified. We have estimated that the repertoire of Class A ORs expressed in the olfactory epithelium in cyprinids to be between 45 and 50 and confirmed this with RT-PCR. Similarly 40–45 TAARs are estimated to be expressed.

Finally we have identified four members of the membrane progestin receptor family. Three of them fit the criteria of possible candidates for main elements in steroid pheromone communication. Namely, they are diverse splice variants of two closely related paralogues of fish maturation inducing-steroid receptors; they demonstrate an olfactory system specific profile of expression in fish; and they are predicted to bind Gαi, which is known to mediate the progestin olfactory response in goldfish. Taken together we suggest that the group of *PAQR5 *and *PAQR6 *gene products could be pheromone receptors in fish, although further studies such as, histochemical and cell culture expression are needed.

## Materials and methods

### Isolation of olfactory epithelia

The goldfish used in this study were obtained from a local pet shop. Fish were anesthetized with MS222 and olfactory tissue was dissected out and placed in liquid nitrogen. Twenty normal body shaped specimens of 10–15 cm and 20–25 g and of both sexes were used.

#### Preparation of a cDNA library

Total RNA was extracted with Tri Reagent and treated with DNAse. mRNA purification and concentration were performed using Dynal's oligo-dT magnetic beads. The normalized olfactory epithelium cDNA library was constructed using cDNA SMART-kit (Clontech) and thermostable duplex-specific nuclease [96]. Sequencing was carried out at the Max Planck Institute of Molecular Genetics (Berlin, Germany) using a Capillary Sequencer systems, ABI 3730 XL (Applied Biosystems) and MegaBase 4500 (GE Healthcare, formally Amersham-Pharmacia) and all sequencing reactions were carried out with ABI BigDye Terminator version 3.1. All the EST sequences were deposited by dbEST in EMBL  under accession numbers AM925430... AM930226.

#### Computational methods

The DNA sequence chromatograms were analyzed using the Phred software [97] for base call and for estimation of error probability at the Max Planck Institute of Molecular Genetics. All sequences were edited to remove ribosomal RNA, polyA tails, low-quality sequences, and vector and adapter regions. Cross-comparison using Liru local blast software was carried out and sequences with more than 95% identity were assembled manually. The resulting contigs and singletons were assessed for similarity to the protein non-redundant database using BlastX at NCBI [98] and sequences annotated if the match complied with the following criteria: e-value < e^-10 ^and > 70% of alignment coverage, or e^-40 ^if only the 3' UTR matched the database. Gene ontology (GO) and Genecards websites were used for functional annotation of identified genes. All annotations and GO assignments were curated manually. To compare GO category counts the WeGo interface was used [99]. The Gene Ontology category selected as a basis for transcriptome size estimation was the Glucose Metabolic Process (GO:0006006). To evaluate the number of orthologous genes present in the library and estimate transcriptome size, a side to side unigene set comparison was performed against zebrafish olfactory epithelium libraries. The libraries used were obtained from NCBI database Unigene [100], dbEST 2387 "Zebrafish adult olfactory" from Washington University School of Medicine and dbEST "NIH_ZGC_14" from National Institutes of Health, Zebrafish Gene Collection. Because none of the libraries were normalized, as indicated in the data annotation, they were pooled together with summation of counts for each unigene present in both of them. All genes, mentioned in the article, were identified from ESTs or amplified by RT-PCR in one or both fish species olfactory epithelium. Alignments were carried out using ClustalW interface at EBI .

#### RT-PCR amplification of olfactory receptors and PAQRs

Total RNA was extracted with Tri Reagent from the olfactory epithelium of 15 goldfish or zebrafish of both sexes at different seasons. Samples were treated with DNAse (Quiagen). Specific primers for each family of GPCR Class A were designed using conserved gene regions identified by multiple alignment of available olfactory receptors from teleost fish species. Primers for PAQR genes were based on sequences from published goldfish sequences (indicated above). Primers used were: mPRγ 12forward-CACACCTTCAGCACCATGTC; mPRγ 12reverse – TCCAGGTGCCAGTCTCTC; mPRαβ forward-TGTGTGGACACACCTGCTGGC; mPRαβ reverse-GTACAGGACAGCCAGGCCAGGA. Amplification was performed using a MyCycler (BioRad) thermocycler with conditions adjusted for each primer pair. The PCR products were cloned in pGem-T-Easy and sequenced in both directions with T7 and SP6 primers using a 3130x sequencer (Applied Biosystems).

## Authors' contributions

NK collected the samples, planned the work, carried out the EST analysis and wrote the manuscript; MK produced and sequenced the goldfish cDNA library; RR contributed with funds and the sequencing platform; AVMC planned the work, provided funds and wrote the manuscript;

## Supplementary Material

Additional file 1 **List of annotated goldfish expressed sequence tags.** The data provided represent the manually curated annotated list of goldfish EST. Click here for file
